# Distinctive alteration in the expression of autophagy genes in Drosophila models of amyloidopathy and tauopathy

**DOI:** 10.1080/03009734.2020.1785063

**Published:** 2020-07-11

**Authors:** Mehrnaz Haghi, Raheleh Masoudi, Seyed Morteza Najibi

**Affiliations:** a Department of Biology, College of Sciences, Shiraz University, Shiraz, Iran; b Center for Molecular Protein Science, Lund University, Lund, Sweden; c Department of Statistics, College of Sciences, Shiraz University, Shiraz, Iran

**Keywords:** Alzheimer’s disease, amyloid beta, autophagy genes, *Drosophila melanogaster*, tau

## Abstract

**Background:**

Alzheimer’s disease (AD) is one the most common types of dementia. Plaques of amyloid beta and neurofibrillary tangles of tau are two major hallmarks of AD. Metabolism of these two proteins, in part, depends on autophagy pathways. Autophagy dysfunction and protein aggregation in AD may be involved in a vicious circle. The aim of this study was to investigate whether tau or amyloid beta 42 (Aβ42) could affect expression of autophagy genes, and whether they exert their effects in the same way or not.

**Methods:**

Expression levels of some autophagy genes, *Hook*, *Atg6*, *Atg8*, and *Cathepsin D*, were measured using quantitative PCR in transgenic *Drosophila melanogaster* expressing either Aβ42 or Tau R406W.

**Results:**

We found that *Hook* mRNA levels were downregulated in Aβ42-expressing flies both 5 and 25 days old, while they were increased in 25-day-old flies expressing Tau R406W. Both *Atg6* and *Atg8* were upregulated at day 5 and then downregulated in 25-day-old flies expressing either Aβ42 or Tau R406W. *Cathepsin D* expression levels were significantly increased in 5-day-old flies expressing Tau R406W, while there was no significant change in the expression levels of this gene in 5-day-old flies expressing Aβ42. Expression levels of *Cathepsin D* were significantly decreased in 25-day-old transgenic flies expressing Tau R406W or Aβ42.

**Conclusion:**

We conclude that both Aβ42 and Tau R406W may affect autophagy through dysregulation of autophagy genes. Interestingly, it seems that these pathological proteins exert their toxic effects on autophagy through different pathways and independently.

## Introduction

Alzheimer’s disease (AD) is characterized by progressive memory impairment and dementia. This neurodegenerative disease is also one of the main causes of death in the elderly population (1). Neurofibrillary tangles (intracellular inclusion of hyperphosphorylated tau) and Aβ plaques (extracellular inclusion of Aβ42 peptide) are two major markers in the brain of AD patients (2). The mechanism underlying AD pathology is not fully understood. Some hypotheses point to these aggregations as the reason for developing AD symptoms. On the other hand, recent investigations have shown that aggregate formation is a defense mechanism against soluble and aggressive forms of Aβ and hyperphosphorylated tau (3,4).

Although these aggregates may exert protection against their soluble oligomer forms, they block axonal/dendritic transport (5) and cause damage to mitochondrial complexes (6,7), leading to increased levels of reactive oxygen species (ROS) in the brain of AD patients. Autophagy (self-eating) is one of the pathways responsible for clearing these aggregations in order to prevent their accumulation (8). To eliminate the aggregates, they are transferred via a dynein/dynactin motor complex to a perinuclear aggresome. Then, they are encapsulated by neurofilaments, and the aggresome is formed. Ultimately, aggregates are cleared when the aggresome is fused to a lysosome (9). Hook protein is an adaptor facilitating the association of motor complex with its cargos (10).

Many stimuli can trigger the initiation of autophagy by ULK1/Atg1 protein kinase complex (11). This serine/threonine kinase phosphorylates Beclin1/Atg6 in the Beclin1-VPS34 PI3 kinase complex involved in nucleation of autophagosome formation (phagophore). Following Atg6 phosphorylation, VPS34 PI3 kinase can convert phosphatidylinositol (PtdIns or PI) to PI 3-phosphate (PI3P), which is essential for expansion of the autophagosome membrane (12,13). Next, elongation phase and phagophore formation occur (14). Two ubiquitin-like pathways, Atg5–Atg12 and the LC3 conjugation systems, are involved in this phase. LC3 (I)/Atg8 is cleaved by Atg4 at the carboxyl-terminal site. Later, LC3 is converted to its active form, LC3II, through covalent bonding to phosphatidyl ethanolamine, which assists the fusion of the autophagosome membrane with the lysosome (15,16). Ultimately, the internal components are degraded by lysosome hydrolases such as Cathepsin D (17–19).

Hyperphosphorylated tau and Aβ aggregates can affect mitochondrial complexes and its electron chain, leading to increased levels of ROS and oxidative stress (6,7). Interestingly, it has been shown that ROS is involved in the induction of autophagy in AD. Lipinski et al. reported that ROS can enhance autophagy through increase in type III PI3 kinase activity. They also confirmed that, unlike in normal aging, autophagy genes are transcriptionally increased in AD patients (20). On the other hand, many studies have shown that ROS can lead to autophagy impairment by various pathways either directly or indirectly. ROS directly inhibits LC3 lipidation and also its translocation to the phagophore membrane (21). ROS increases NF-κB and nitric oxide (NO) synthase which, in turn, enhances NO levels (22). NO can inhibit JNK1 via *S*-nitrosylation at C116, leading to reduction of Bcl2 phosphorylation, which ultimately increases its interaction with Beclin1. This event disrupts the formation of the hVps34/Beclin1 complex, and autophagy is eventually impaired (23).

It seems that presence of protein aggregates and autophagy dysfunction in AD create a vicious circle. The aim of this study was to investigate the effect of pathological Tau R406W and Aβ42, two major causes of aggregate formation in AD, on the expression of autophagy genes, *Atg6*, *Atg8*, *Hook*, and *Cathepsin D*. These genes are involved in different stages of autophagy. Considering that AD is an age-dependent disorder, the expression levels of autophagy genes were assessed at two different time points in Aβ42 or Tau R406W transgenic *Drosophila melanogaster*. Tau R406W is an autosomal dominant mutation that causes tau-positive frontotemporal dementia in human. Its expression in *Drosophila melanogaster*, using a binary system, provides a proper tauopathy model (24).

## Materials and methods

### Chemicals

RNA extraction and cDNA synthesis kits were purchased from CinnaGen and Parstous companies, Iran, respectively. Brilliant II SYBR Green qPCR master mix was provided by Biofact, Germany. Other materials used in this study were provided from CinnaGen, Iran and Merck, Germany.

### Fly strains

*Drosophila melanogaster* stocks were raised in standard rolled oats-agar medium at 22 ± 1 °C, 60–70% humidity, and 12-h light/12-h dark circadian cycle. MAPT R406W for tauopathy (expression of this protein in transgenic fly is discussed in Wittmann et al., in 2001) (24) and Aβ42 for amyloidopathy (Bloomington Stock No. 33769) (25) were expressed in neurons using Dmel\P{GawB}elavC155 (Bloomington Stock No. 458) driver. While expressed tau remains in the cytoplasm, presence of a signal peptide in the Aβ42 construct (26) causes Aβ42 transportation to the endoplasmic reticulum (27). To investigate the pathogenesis of Aβ42 and Tau R406W, GMR-Gal4 driver (Bloomington Stock No. 8605) (28) was applied to express these proteins in the fly eyes.

Flies expressing UAS-Tau R406W were from Feany’s lab (Harvard Medical School, Boston, MA, USA), and all other lines were from Bloomington Drosophila Stock Centre. All crosses and their counterpart controls (parental lines) were applied in triplicate. All transgenic stocks were outcrossed to w^1118^ for several generations to obtain the identical genetic background of all lines, prior to the tests.

### Climbing assay

For climbing assay analysis, nine groups of flies, with 10 flies per group (mix of both genders), were prepared for every single genotype. All 10 flies were transferred into a vial, and the vial was tapped gently. After each tap, flies were observed for the first 10 s to record the number of flies that were able to climb above the 8-cm marked line on the vial. This assay was repeated five times for each group with a 2-min interval between each measurement (29,30).

### Drosophila eye analysis

Transgenic (GMR-Gal4/Tau R406W and GMR-Gal4/Aβ42) and control (GMR-Gal4/+) flies from at least three independent crosses (9 flies per group, per cross) were processed for light microscopy and image analysis with three experimental repeats.

Imaging was performed using a Nikon 80i light microscope to observe degeneration phenotype in the fly eyes. A new plugin (FLEYE) in ImageJ software was used to analyze the fly eyes. Regularity in the ommatidia was represented by probability parameter (PP) including: PP0, green; PP1, blue; PP2, yellow; PP3, orange; and finally PP4, red. Change in the colour from green to red represents the degree of reduction in eye regularity (28).

### Quantitative real-time PCR

First filial generation (F1) and their parental lines (as control) were collected at 5 and 25 days after eclosion. For RNA extraction, flies were kept in acetone and stored in a −20 °C freezer overnight (31,32). Then, flies were frozen at −80 °C. After 10 min, flies were shaken harshly in the tube in order to separate the heads. RNA was extracted from 100 heads on liquid nitrogen by RNX plus kit following the manufacturer’s protocol. RNA concentration was measured using Nano drop (Thermo Fisher Scientific), and 3 µg of total RNA was reverse-transcribed to cDNA. Amplification of cDNA was performed using Biofact SYBER Green master mix in an ABI 7500 PCR machine. All primers were designed as the exon–exon junction primer. *RPL32* was applied as reference gene to determine the relative expression levels of *Hook*, *Atg6*, *Atg8*, and *Cathepsin D* using a 2(-delta delta C(T)) method (33). Each sample was run in triplicate. The sequences of primers are given in Supplementary Table S1.

### Statistical analysis

Here, we applied R (version 3.6.1) and SPSS (version 19) programmes to perform statistical analysis.

**Figure 1.**
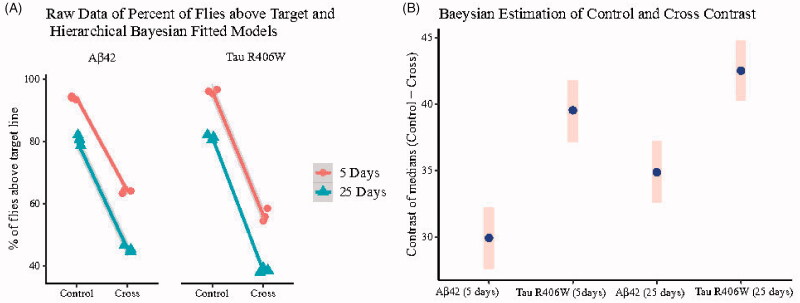
(A) Representing the raw data of the percent of flies (control and cross) above the target line in three independent biological repeats for 5- and 25-day-old flies and the fitted Bayesian hierarchical model lines for each group and subgroup and their shaded 95% highest posterior density (HPD) region based on 2000 posterior samplings of model parameters. (B) Bayesian estimation of contrast between medians (control–cross) and their 95% HPD region based on 2000 posterior samplings of each contrast. Because zero is not included in any of the reported HPD’s intervals, there is significant difference between the control and cross negative geotaxis ability with 5% error type (*α* = 0.05).

**Figure 2.**
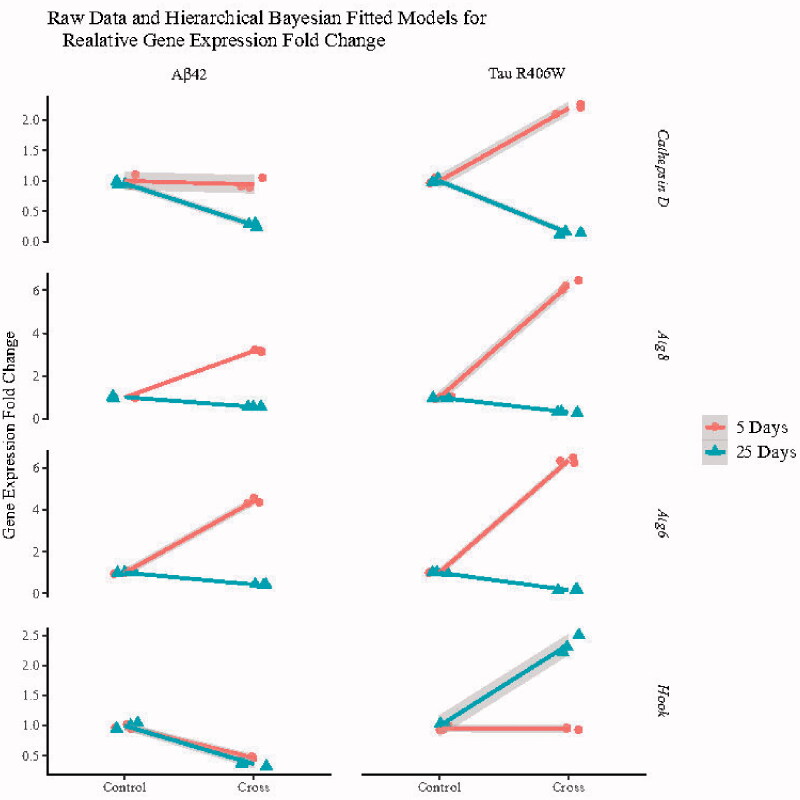
Representing the raw data of autophagy-related gene expression in three independent biological repeats of Aβ42 or Tau R406W transgenic flies and the fitted Bayesian hierarchical model lines for each group and subgroup shaded by their 95% HPD region based on 2000 posterior samplings of model parameters.

The number of samples in biological studies is usually small, and the assumptions of parametric statistical models cannot be achieved. One solution is to use alternative non-parametric statistical models, which have lower power compared to the parametric versions. Another solution is to use Bayesian inference, which is more powerful to infer reliable and reproducible results (34–36). In this study, because Aβ42 and Tau R406W have two subgroups (cross and control), we used the Bayesian hierarchical mixture model for testing. Our hypotheses were that there are differential gene expression and climbing ability in two biologic conditions (AD and control) at different time points (5 and 25 days). The proposed model exploits available position-specific read counts, minimizing required data pre-processing and making maximum use of available information. Our analysis has been done by Stan language that is using a state-of-the-art algorithm known as Hamiltonian Monte Carlo (HMC), which builds upon the Metropolis–Hastings algorithm by incorporating many theoretical ideas from physics. Specifically, we used the *rstanarm* package in *R* software, which is a powerful package for Bayesian hierarchical models by *stan_lme4* for estimating the model parameters. In this paper, we considered a full multilevel model for group/subgroup*time*(genes) that means we assume random intercept and slope for each category. We had to have an estimation of different intercepts and slopes for each experimental condition, which was clearly needed based on the raw data (see Figures 1 and 2). The number of samples is selected to be 4000 (2000 for warm-up), and four independent chains ran for the sake of convergency and posterior sampling evaluation. The median estimation and the highest posterior density (HPD) intervals with probability of 0.95 are reported based on 2000 samples from posterior distribution of model parameters. The Bayesian contrast estimations are evaluated by the *emmeans* package in R.

Besides the Bayesian model, we have also presented our data as fold changes when comparing crosses and their counterpart controls. Independent samples *t* test was employed to compare the mean values. *p* values less than 0.05 were considered as statistically significant.

## Results

### Decrease in the climbing ability of Aβ42 or Tau R406W transgenic flies

Locomotive defects can be examined in transgenic flies as one of the AD symptoms. Here, we tested negative geotaxis ability as a behavioural assay to show that the expression of our target genes can affect the natural tendency of flies to move against gravity (29,30). To assess the climbing ability, 5- and 25-day-old transgenic flies were examined. It was found that the ability of climbing in both transgenic flies was remarkably decreased compared to their controls. The raw data and Bayesian estimation of difference between controls and transgenic flies are depicted in Figure 1 (for more details see Table 1; and also Supplementary Table S2).

**Table 1.** Bayesian contrast estimation of medians (control–cross) for climbing assay in 5- and 25-day-old flies.

Time point	Fly type	Contrast estimate (control–cross)	Lower HPD (2.5%)	Upper HPD (97.5%)
5 days	Aβ42	29.92^a^	27.55	32.12
	Tau R406W	39.49^a^	37.13	41.67
25 days	Aβ42	34.83^a^	32.44	37.04
	Tau R406W	42.53^a^	40.15	44.74

^a^ The 95% highest posterior density (HPD) intervals for each contrast are reported. Therefore, there is a significant difference between control and cross with 5% error type I (*α* = 0.05) in all of the cases thus marked.

There was 0.32- and 0.42-fold decrease (*p* values ≤ 0.001) in 5-day-old Aβ42 or Tau R406W-expressing flies, respectively; 25-day-old flies expressing Aβ42 or Tau R406W showed 0.44- and 0.53-fold reduction (*p* values ≤0.001) in the climbing ability, respectively. For more details see Supplementary Figure S1 and Supplementary Table S3.

### Eye degeneration was observed in flies expressing either Tau R406W or Aβ42

**Figure 3.**
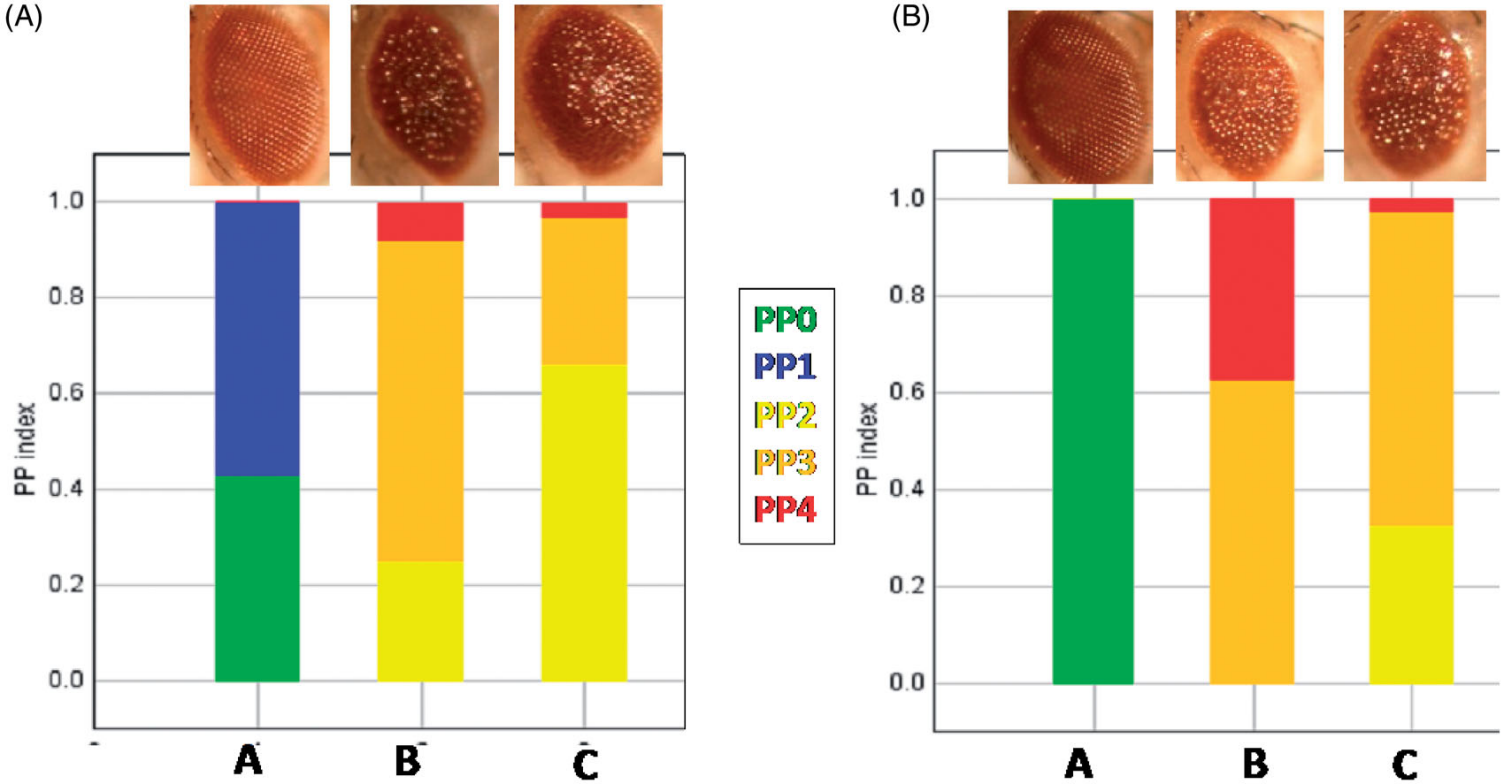
Eye degeneration has been observed in flies expressing Tau R406W or Aβ42. A and B, respectively, are analyses for eye ommatidium regularity in 5- and 25-day-old flies by ImageJ, and columns A, B, and C are GMR-GAL4/+, GMR-GAL4/Tau R406W, and GMR-GAL4/Aβ42. (PP = probability parameter)

To screen the pathogenesis of our genes of interest (Aβ42 and Tau R406W), we investigated the degeneration of Drosophila retina as a model system by expressing the transgenes using the GMR-Gal4 driver. As can be seen in Figure 3, there was more irregularity (yellow and red colors) in the eye ommatidia of transgenic flies compared to parental lines with regular eye ommatidia (blue and green colour). Interestingly, both Aβ42 and Tau R406W transgenic flies demonstrate irregularities in their eyes.

### Quantitative real time PCR

#### Alteration in the expression of Hook, as a pre-autophagy gene

**Figure 4.**
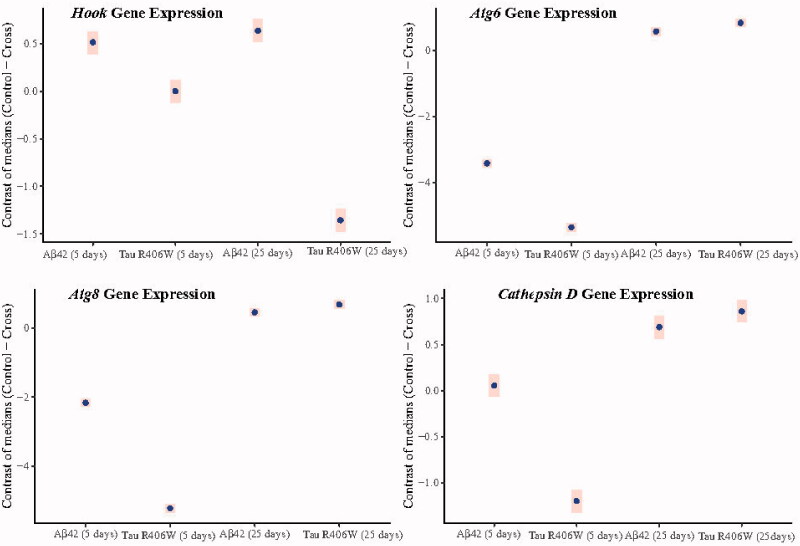
Bayesian estimation of median contrasts (control–cross) for gene expression in 5- and 25-day-old flies. *Hook* gene has different expression pattern in Aβ42- or Tau R406W-expressing flies either 5 or 25 days old, while *Atg6* and *Atg8* mRNA levels changed with similar pattern in both transgenic lines either 5 or 25 days after eclosion.

As mentioned earlier, *Hook* is a mediator to facilitate interaction between motor proteins and their cargo as a pre-autophagy protein (37). In the AD brain, reduction in *Hook*3 expression and increase in *Hook*2 mRNA levels have been observed. Interestingly, when *Hook*3 is knocked down, there is an increase in Aβ production (38). While there are three forms of *Hook* in humans, only one form of *Hook* has been reported in Drosophila. Here, we assessed the effect of Aβ42 and Tau R406W on the expression levels of *dHook* (Drosophila *Hook*) using transgenic flies. As can be seen in Figure 2 and from Bayesian contrast estimations in Table 2, although there was no significant change in the levels of *dHook* expression in 5-day-old flies expressing Tau R406W, a prominent increase was observed in the levels of this gene in 25-day-old flies. It seems that Tau R406W exerts its effect on the *Hook* expression at a later time point of the life cycle of this fly. Regarding Aβ42-expressing flies, there was a significant decrease in the levels of *Hook* expression in both 5- and 25-day-old flies. For more details see Figure 4; and also Supplementary Table S4.

**Table 2.** Bayesian contrast estimation for medians of genes expressions (control–cross) in 5- and 25-day-old flies and different genes and fly types.

Gene type	Time point	Fly type	Contrast estimate (control–cross)	Lower HPD (2.5%)	Upper HPD (97.5%)
*Hook*	5 days	Aβ42	0.512^a^	0.3964	0.625
		Tau R406W	−0.0002	−0.114	0.128
	25 days	Aβ42	0.647^a^	0.525	0.764
		Tau R406W	−1.354^a^	−1.475	−1.235
*Atg6*	5 days	Aβ42	−3.414^a^	−3.530	−3.296
		Tau R406W	−5.353^a^	−5.470	−5.231
	25 days	Aβ42	0.569^a^	0.444	0.681
		Tau R406W	0.829^a^	0.716	0.951
*Atg8*	5 days	Aβ42	−2.164^a^	−2.290	−2.046
		Tau R406W	−5.229^a^	−5.343	−5.106
	25 days	Aβ42	0.452^a^	0.335	0.567
		Tau R406W	0.685^a^	0.567	0.803
*Cathepsin D*	5 days	Aβ42	0.058	−0.065	0.173
		Tau R406W	−1.196^a^	−1.309	−1.078
	25 days	Aβ42	0.683^a^	0.564	0.800
		Tau R406W	0.858^a^	0.743	0.977

^a^ The 95% highest posterior density (HPD) intervals for each contrast are reported. Therefore, there is a significant difference between control and cross with 5% error type I (*α* = 0.05) in all of the cases thus marked.

Regarding differential expression, there was about a 2.26-fold increase in the *Hook* expression in 25-day-old flies expressing Tau R406W (*p* = 0.018). The reduction in the expression of *Hook* was around 0.5- and 0.59-fold for 5- and 25-day-old Aβ42-expressing flies, respectively (*p* = 0.001 and 0.002). More details are in the Supplementary data, Supplementary Figure S2 and Supplementary Table S5.

#### Alteration in the expression of autophagy genes

Expression of autophagy genes in fly is influenced by various factors like age (39) and oxidative stress (40). On the other hand, it has been shown that suppression or enhancement of some autophagy genes like *Atg8* can increase the sensitivity to aging and oxidative stress (41). Here, the expression levels of three autophagy markers including *Atg6*, *Atg8*, and *Cathepsin D* were assessed to track the autophagy process in Drosophila models of AD. Transgenic flies expressing either Tau R406W or Aβ42 were applied as tauopathy or amyloidopathy models at two different ages, 5- and 25-day-old flies. This could provide some information on how time might affect the expression of some autophagy genes in the presence of two different types of amyloid-like aggregates.

### Atg6, a core protein in the nucleation stage of autophagy

One of the main proteins for phagophore formation is Atg6. According to the Bayesian model analysis, our data showed (Figure 2 and Table 2) that *Atg6* was upregulated in 5-day-old flies expressing either Aβ42 or Tau R406W while the mRNA levels of this gene had a considerable decrease in 25-day-old transgenic flies (for more details, see Figure 4; and also Supplementary Table S4). It seems that both Tau R406W and Aβ42 aggregates exert the same effect on the expression levels of this gene at different time points.

There was a 4.45- and 6.31-fold increase in the levels of *Atg6* in 5-day-old flies expressing Aβ42 or Tau R406W, respectively (*p* ≤ 0.001). In contrast, 25 days after eclosion these transgenic flies showed a significant decrease (*p* ≤ 0.001), around 0.58- and 0.85-fold, in the *Atg6* expression levels compared to their counterpart controls. For more details see Supplementary Figure S2 and Supplementary Table S6.

### Atg8, as the main gene in the elongation phase

Atg8 is the marker protein in the elongation phase of the autophagosome formation. This unique protein undergoes two processing steps. In the first step, Atg8 is cleaved by Atg4 and the next step is mediated by the Atg5–Atg12–Atg16 complex to get conjugated with phosphatidylethanolamine. These two steps are necessary for elongation and closure of the autophagic membrane (42). The expression of this gene showed the same pattern as the *Atg6*. There was a significant increase in the expression levels of *Atg*8 in 5-day-old transgenic flies expressing either Tau R406W or Aβ42, followed by a prominent decline in the levels of *Atg*8 mRNA expression in those flies 25 days after eclosion (for more details on the Bayesian model see Figures 2 and 4, and Table 2; and also Supplementary Table S4). Therefore, it seems that both forms of aggregates have similar impact on the *Atg*8 mRNA levels.

In the fold change analysis, 5-day-old Aβ42 or Tau R406W transgenic flies showed a 3.15- and 6.28-fold increase in the levels of *Atg*8 (*p* ≤ 0.001), while there was a significant decline (*p* ≤ 0.001) in the expression levels of this gene (about 0.45- and 0.70-fold, respectively) in 25-day-old flies expressing Aβ42 or Tau R406W (See Supplementary Figure S2 and Supplementary Table S7 for more details).

### Expression of the lysosome enzyme Cathepsin D, involved in the final step of autophagy

In the central nervous system, the activity of Cathepsin D is essential to control neuronal homeostasis, cell migration, and interneuron communication. Cathepsin D-mediated proteolysis plays a significant role in neuronal survival by accomplishing the degradation of aggregated proteins that reach the lysosomes via autophagy (43). There is evidence to reveal that this protein is involved in amyloidogenic processing of the amyloid precursor protein (APP), as a critical component of α, β, and γ secretase (44). We found that the mRNA levels of Cathepsin D have different patterns in 5-day-old flies expressing Tau R406W or *Aβ*42. According to the Bayesian model, there was no significant change in the levels of Cathepsin D mRNA in flies expressing *Aβ42*, while Tau R406W-expressing flies showed a prominent increase in the mRNA levels of this gene at day 5 after eclosion. Interestingly, at day 25, both transgenic flies showed a remarkable decline in the expression levels of Cathepsin D mRNA (Figures 2 and 4, and Table 2; and also Supplementary Table S4).

The fold change analysis showed that there was a 2.24-fold increase (*p* = 0.007) in the levels of Cathepsin D expression in 5-day-old flies expressing Tau R406W. In 25-day-old Aβ42 or Tau R406W-expressing flies, there was a 0.74- and 0.85-fold decrease, respectively (*p* ≤ 0.001). More details are included in Supplementary Figure S2 and Supplementary Table S8.

## Discussion

According to the amyloid cascade, proposed by Hardy and Higgins in 1992, amyloid is the main culprit in AD pathology (44). Other events in AD, including tau hyperphosphorylation and subsequent neuronal death, were supposed to be downstream events of the amyloid pathway (45–47). Therefore, most treatments for AD have been based on the removal of Aβ (reviewed in 46). Recently, there is increasing evidence to support the idea that pathological tau can exert its own toxic effect independently (48–50). On the other hand, there are some studies showing that aggregations of Aβ (senile plaque, SP) and tau (neurofibrillary tangles, NFT) are, in fact, a defensive mechanism against Aβ monomers and the soluble pathological tau (3,51). However, these aggregates, in turn, block axonal/dendritic transport (5) and can lead to mitochondrial damage and an increase in the levels of ROS (6,7).

In order to shed light on the mechanism underlying the relation between protein aggregates and autophagy dysfunction in AD, here, we investigated the differential expression of autophagy genes in Aβ42-expressing flies as an amyloidopathy model and flies expressing Tau R406W as a tauopathy model (24). We used Tau R406W transgenic flies as they have been reported to exhibit AD-like phenotypes (52). On the other hand, previous studies have shown that tau wild-type transgenic flies have no aggregate formation (53). Therefore, to clarify the effect of the aggregates of this protein on autophagy genes, this model was more suitable.

Our climbing assay and fly eye analysis showed that Aβ42 and Tau R406W can cause defects in climbing ability and eye regularity in transgenic flies. These data also showed that tau can exert its toxicity independent of Aβ42.

Neurons are highly dependent on the autophagy to remove protein aggregates such as SP and NFT (8). Defects in this pathway clearly lead to neurodegenerative disorders such as AD. There is strong evidence to confirm the association of autophagy dysregulation with AD (54). Herrmann and colleagues showed that Hook3 was co-localized with tau aggregate and retained in those aggregates (38). Surprisingly, in our current research, there was a remarkable increase in the levels of *dHook* expression in 25-day-old flies expressing Tau R406W. It is possible that Hook is the adaptor for tau aggregates in the autophagy pathway and that is why the expression of *Hook* is increased in Tau R406W-expressing flies. However, the expression of this gene is downregulated in Aβ42-expressing flies. This may suggest that either the transportation of Aβ aggregates is mainly managed by another adaptor or Aβ42 can affect autophagy through *Hook* downregulation. In summary, it seems that although both Tau R406W and Aβ42 alter the expression of *Hook*, their effects occur via different mechanisms.

Several studies have shown that Beclin1 (ATG6), a main protein to initiate autophagy, is downregulated in AD, while some other evidence argues that autophagy genes are increased during AD (20,55,56). Despite all the controversies, it is clear that autophagy is deregulated in this disease.

Atg6 is downregulated in early and late stages of AD (55). However, it has been shown that *ATG8* expression is increased in AD patients (56,57). According to our results, while *Atg6* and *Atg8* showed upregulation in their mRNA levels in 5-day-old flies expressing either Tau R406W or Aβ42, a remarkable downregulation was observed for both genes at day 25 after eclosion. It appears that in earlier stages (5 day after eclosion) autophagy genes are increased in order to clear the aggregates. However, ultimately autophagy is decreased at later time points, probably due to an increase in the ROS production (6,7) or other mechanisms.

Association of autophagosome with lysosome is the last step of autophagy. Finally, its internal components are degraded by lysosome hydrolases like Cathepsin D (17). Cathepsin D is the only proteolytic enzyme the expression of which, in different tissues, is regulated in response to growth factors, cytokines, and vitamins (58). Cathepsin D-mediated proteolysis is essential to neurons because it degrades unfolded/oxidized protein aggregates that continuously reach the lysosomes via autophagy or endocytosis (43). Many proteins produced in neurons are physiologic substrates of Cathepsin D and will be abnormally accumulated if they are not efficiently degraded (e.g. APP, α-synuclein, and huntingtin). Therefore, dysfunction of Cathepsin D in the lysosomal system is closely related to the mechanism underpinning neurodegeneration (43).

In 2006, Urbanelli et al. demonstrated that there was a decrease in the *Cathepsin D* mRNA and protein levels in AD (59). However, in 1995, Cataldo et al. demonstrated intense Cathepsin D immunoreactivities and lowered *Cathepsin D* mRNA levels in degenerating neurons (60). The upregulation of *Cathepsin D* synthesis and accumulation of hydrolase-laden lysosomes indicated an early activation of the endosomal-lysosomal system in vulnerable neuronal populations, possibly reflecting early regenerative or repair processes (60). Urbanelli and colleagues provided evidence of altered balance of the *Cathepsin D* expression in skin fibroblasts from patients with sporadic or familial forms of AD (59). In particular, they showed that the expression of this gene is downregulated at both the transcriptional and translational levels and its processing is altered in AD fibroblasts (59). High levels of the constitutively active form of Ras in normal or AD fibroblasts induce *Cathepsin D* downregulation. Furthermore, the p38 MAPK signalling pathway also appears to downregulate the *Cathepsin D* levels. Urbanelli et al. proposed that the impairment of lysosomes in AD can be one of the main factors for the progression of the disease (59).

Finally, in 2018 Chai et al. investigated the Cathepsin D immunoreactivities in the temporal and parietal cortices of well characterized AD brains. Their results showed an increase in the Cathepsin D immunoreactivities in AD tissues and its correlation with neuropathological NFT scores, and phosphorylated pSer396 tau burden (17).

Our data showed that there was a significant increase in the mRNA levels of *Cathepsin D* in Tau R406W-expressing flies just 5 days after eclosion. This increase could be due to regenerative or repair processes occurring at earlier stages of the disease (60). However, in 25-day-old transgenic flies, this gene showed significant reduction. Following the increase in Tau R406W and Aβ42 aggregations at later time points (25-day-old flies), probably due to an increase in ROS production, *Cathepsin D* is downregulated (43). Moreover, cystatin C, as an inhibitor of cathepsins (cysteine protease), is upregulated by ROS and has been shown to be co-localized with the Aβ42 peptide (61,62).

## Conclusion

In summary, our results suggest that both Aβ42 and Tau R406W can affect the autophagy pathway through gene expression dysregulation. Interestingly, they showed a similar effect on the genes involved in the nucleation and elongation steps of autophagy. However, Tau R406W and Aβ42 exert different effects on the expression of the pre-autophagy gene, *Hook*, and a gene involved in the last step of autophagy, *Cathepsin D*. We conclude that although both Tau R406W and Aβ42 can alter the process of autophagy during AD, it seems that they act independently, through different mechanisms. Therefore, AD treatment involving the removal of merely Aβ42, without considering pathological tau, will not be sufficiently effective in ameliorating AD symptoms.

## Supplementary Material

Supplemental Material

## References

[1] Hayashi SI, Sato N, Yamamoto A, Ikegame Y, Nakashima S, Ogihara T, et al Alzheimer disease-associated peptide, amyloid beta40, inhibits vascular regeneration with induction of endothelial autophagy. Arterioscler Thromb Vasc Biol. 2009;29:1909–15. 19815818 10.1161/ATVBAHA.109.188516

[2] Caccamo A, Maldonado MA, Majumder S, Medina DX, Holbein W, Magrí A, et al Naturally secreted amyloid-beta increases mammalian target of rapamycin (mTOR) activity via a PRAS40-mediated mechanism. J Biol Chem. 2011;286:8924–32. 21266573 10.1074/jbc.M110.180638 PMC3058958

[3] Esparza TJ, Gangolli M, Cairns NJ, Brody DL. Soluble amyloid-beta buffering by plaques in Alzheimer disease dementia versus high-pathology controls. PLoS One. 2018;13:e0200251. 29979775 10.1371/journal.pone.0200251 PMC6034844

[4] Götz J, Ittner LM, Fändrich M, Schonrock N. Is tau aggregation toxic or protective: a sensible question in the absence of sensitive methods? J Alzheimers Dis. 2008;14:423–9. 18688093 10.3233/jad-2008-14410

[5] Bendiske J, Bahr BA. Lysosomal activation is a compensatory response against protein accumulation and associated synaptopathogenesis-an approach for slowing Alzheimer disease? J Neuropathol Exp Neurol. 2003;62:451–63. 12769185 10.1093/jnen/62.5.451

[6] Golpich M, Amini E, Mohamed Z, Azman Ali R, Mohamed Ibrahim N, Ahmadiani A. Mitochondrial dysfunction and biogenesis in neurodegenerative diseases: pathogenesis and treatment. CNS Neurosci Ther. 2017;23:5–22. 27873462 10.1111/cns.12655 PMC6492703

[7] Schulz KL, Eckert A, Rhein V, Mai S, Haase W, Reichert AS, et al. A new link to mitochondrial impairment in tauopathies. Mol Neurobiol. 2012;46:205–16. 22847631 10.1007/s12035-012-8308-3

[8] Uddin M, Stachowiak A, Mamun AA, Tzvetkov NT, Takeda S, Atanasov AG, et al. Autophagy and Alzheimer’s disease: from molecular mechanisms to therapeutic implications. Front Aging Neurosci 2018;10:4. 10.3389/fnagi.2018.00004 PMC5797541 29441009

[9] Chin LS, Olzmann JA, Li L. Aggresome formation and neurodegenerative diseases: therapeutic implications. Curr Med Chem. 2008;15:47–60. 18220762 10.2174/092986708783330692 PMC4403008

[10] Olenick MA, Tokito M, Boczkowska M, Dominguez R, Holzbaur EL. Hook adaptors induce unidirectional processive motility by enhancing the dynein-dynactin interaction. J Biol Chem. 2016;291:18239–51. 27365401 10.1074/jbc.M116.738211 PMC5000072

[11] Hurley JH, Young LN. Mechanisms of autophagy initiation. Annu Rev Biochem. 2017;86:225–44. 28301741 10.1146/annurev-biochem-061516-044820 PMC5604869

[12] Fitzwalter BE, Thorburn A. Recent insights into cell death and autophagy. Febs J. 2015;282:4279–88. 26367268 10.1111/febs.13515 PMC4885685

[13] Shravage BV, Hill JH, Powers CM, Wu L, Baehrecke EH. Atg6 is required for multiple vesicle trafficking pathways and hematopoiesis in Drosophila. Development. 2013;140:1321–9. 23406899 10.1242/dev.089490 PMC3585664

[14] Reggiori F, Ungermann C. Autophagosome maturation and fusion. J Mol Biol. 2017;429:486–96. 28077293 10.1016/j.jmb.2017.01.002

[15] Badadani M. Autophagy mechanism, regulation, functions, and disorders. ISRN Cell Biol. 2012;2012:1–11.

[16] Barth S, Glick D, Macleod KF. Autophagy: assays and artifacts. J Pathol. 2010;221:117–24. 20225337 10.1002/path.2694 PMC2989884

[17] Chai YL, Chong JR, Weng J, Howlett D, Halsey A, Lee JH, et al Lysosomal cathepsin D is upregulated in Alzheimer's disease neocortex and may be a marker for neurofibrillary degeneration. Brain Pathol. 2019;29:63–74. 30051532 10.1111/bpa.12631 PMC8028263

[18] Kuchitsu Y, Fukuda M. Revisiting Rab7 functions in mammalian autophagy: Rab7 knockout studies. Cells 2018;7:215. 10.3390/cells7110215 PMC6262614 30463228

[19] Fujita N, Huang W, Lin TH, Groulx JF, Jean S, Nguyen J, et al. Genetic screen in Drosophila muscle identifies autophagy-mediated T-tubule remodeling and a Rab2 role in autophagy. Elife. 2017;6:e23367. 28063257 10.7554/eLife.23367 PMC5249261

[20] Lipinski MM, Zheng B, Lu T, Yan Z, Py BF, Ng A, et al Genome-wide analysis reveals mechanisms modulating autophagy in normal brain aging and in Alzheimer's disease. Proc Natl Acad Sci USA. 2010;107:14164–9. 20660724 10.1073/pnas.1009485107 PMC2922576

[21] Burgoyne JR. Oxidative stress impairs autophagy through oxidation of ATG3 and ATG7. Autophagy 2018;14:1092–3. 29746182 10.1080/15548627.2018.1444311 PMC6103406

[22] Kaur U, Banerjee P, Bir A, Sinha M, Biswas A, Chakrabarti S. Reactive oxygen species, redox signaling and neuroinflammation in Alzheimer's disease: the NF-κB connection. Curr Top Med Chem. 2015;15:446–57. 25620241 10.2174/1568026615666150114160543

[23] Sarkar S, Korolchuk VI, Renna M, Imarisio S, Fleming A, Williams A, et al. Complex inhibitory effects of nitric oxide on autophagy. Mol Cell. 2011;43:19–32. 21726807 10.1016/j.molcel.2011.04.029 PMC3149661

[24] Wittmann CW, Wszolek MF, Shulman JM, Salvaterra PM, Lewis J, Hutton M, et al. Tauopathy in Drosophila: neurodegeneration without neurofibrillary tangles. Science. 2001;293:711–4. 11408621 10.1126/science.1062382

[25] Tare M, Modi RM, Nainaparampil JJ, Puli OR, Bedi S, Fernandez-Funez P, et al. Activation of JNK signaling mediates amyloid-ss-dependent cell death. PLOS One. 2011;6:e24361. 21949710 10.1371/journal.pone.0024361 PMC3173392

[26] Chouhan AK, Guo C, Hsieh YC, Ye H, Senturk M, Zuo Z, et al. Uncoupling neuronal death and dysfunction in Drosophila models of neurodegenerative disease. Acta Neuropathol Commun. 2016;4:62. 27338814 10.1186/s40478-016-0333-4 PMC4918017

[27] Finelli A, Kelkar A, Song HJ, Yang H, Konsolaki M. A model for studying Alzheimer's Abeta42-induced toxicity in *Drosophila melanogaster*. Mol Cell Neurosci. 2004;26:365–75. 15234342 10.1016/j.mcn.2004.03.001

[28] Diez-Hermano S, Valero J, Rueda C, Ganfornina MD, Sanchez D. An automated image analysis method to measure regularity in biological patterns: a case study in a Drosophila neurodegenerative model. Mol Neurodegener. 2015;10:9. 25887846 10.1186/s13024-015-0005-z PMC4367968

[29] Madabattula ST, Strautman JC, Bysice AM, O’Sullivan JA, Androschuk A, Rosenfelt C. Quantitative analysis of climbing defects in a Drosophila model of neurodegenerative disorders. J Vis Exp 2015;100:e52741. 10.3791/52741 PMC4544889 26132637

[30] Ali YO, Escala W, Ruan K, Zhai RG. Assaying locomotor, learning, and memory deficits in Drosophila models of neurodegeneration. J Vis Exp 2011;49:2504. 10.3791/2504 PMC3197301 21445036

[31] Koopmans M, Monroe SS, Coffield LM, Zaki SR. Optimization of extraction and PCR amplification of RNA extracts from paraffin-embedded tissue in different fixatives. J Virol Methods. 1993;43:189–204. 8396155 10.1016/0166-0934(93)90076-4 PMC7119522

[32] Fukatsu T. Acetone preservation: a practical technique for molecular analysis. Mol Ecol. 1999;8:1935–45. 10620236 10.1046/j.1365-294x.1999.00795.x

[33] Livak KJ, Schmittgen TD. Analysis of relative gene expression data using real-time quantitative PCR and the 2(-delta delta C(T)) method. Methods. 2001;25:402–8. 11846609 10.1006/meth.2001.1262

[34] Austin PC, Brunner LJ, Hux JE. Bayeswatch: an overview of Bayesian statistics. J Eval Clin Pract. 2002;8:277–86. 12060417 10.1046/j.1365-2753.2002.00338.x

[35] Button KS, Ioannidis JP, Mokrysz C, Nosek BA, Flint J, Robinson ES, et al. Power failure: why small sample size undermines the reliability of neuroscience. Nat Rev Neurosci. 2013;14:365–76. 23571845 10.1038/nrn3475

[36] R Core Team. R: A language and environment for statistical computing. Vienna, Austria: R Foundation for Statistical Computing; 2020 Available at: http://www.R-project.org/.

[37] Szebenyi G, Hall B, Yu R, Hashim AI, Krämer H. Hook2 localizes to the centrosome, binds directly to centriolin/CEP110 and contributes to centrosomal function. Traffic. 2007;8:32–46. 17140400 10.1111/j.1600-0854.2006.00511.x

[38] Herrmann L, Wiegmann C, Arsalan-Werner A, Hilbrich I, Jäger C, Flach K, et al. Hook proteins: association with Alzheimer pathology and regulatory role of hook3 in amyloid beta generation. PLOS One. 2015;10:e0119423. 25799409 10.1371/journal.pone.0119423 PMC4370497

[39] Rubinsztein DC, Shpilka T, Elazar Z. Mechanisms of autophagosome biogenesis. Curr Biol. 2012;22:R29–R34. 22240478 10.1016/j.cub.2011.11.034

[40] Simonsen A, Cumming RC, Brech A, Isakson P, Schubert DR, Finley KD. Promoting basal levels of autophagy in the nervous system enhances longevity and oxidant resistance in adult Drosophila. Autophagy 2008;4:176–84. 18059160 10.4161/auto.5269

[41] Wu H, Wang MC, Bohmann D. JNK protects Drosophila from oxidative stress by trancriptionally activating autophagy. Mech Dev. 2009;126:624–37. 19540338 10.1016/j.mod.2009.06.1082 PMC2750887

[42] Shpilka T, Weidberg H, Pietrokovski S, Elazar Z. Atg8: an autophagy-related ubiquitin-like protein family. Genome Biol. 2011;12:226. 21867568 10.1186/gb-2011-12-7-226 PMC3218822

[43] Di Domenico F, Tramutola A, Perluigi M. Cathepsin D as a therapeutic target in Alzheimer's disease. Expert Opin Ther Targets. 2016;20:1393–5. 27805462 10.1080/14728222.2016.1252334

[44] Straface E, Matarrese P, Gambardella L, Vona R, Sgadari A, Silveri MC, et al. Oxidative imbalance and cathepsin D changes as peripheral blood biomarkers of Alzheimer disease: a pilot study. FEBS Lett. 2005;579:2759–66. 15907478 10.1016/j.febslet.2005.03.094

[45] Hardy JA, Higgins GA. Alzheimer's disease: the amyloid cascade hypothesis. Science. 1992;256:184–5. 1566067 10.1126/science.1566067

[46] Lewis J, Dickson DW, Lin WL, Chisholm L, Corral A, Jones G, et al. Enhanced neurofibrillary degeneration in transgenic mice expressing mutant tau and APP. Science. 2001;293:1487–91. 11520987 10.1126/science.1058189

[47] Rajmohan R, Reddy PH. Amyloid-beta and phosphorylated tau accumulations cause abnormalities at synapses of Alzheimer's disease neurons. J Alzheimers Dis. 2017;57:975–99. 27567878 10.3233/JAD-160612 PMC5793225

[48] Kametani F, Hasegawa M. Reconsideration of amyloid hypothesis and tau hypothesis in Alzheimer's Disease. Front Neurosci. 2018;12:25. 29440986 10.3389/fnins.2018.00025 PMC5797629

[49] Belrose JC, Masoudi R, Michalski B, Fahnestock M. Increased pro-nerve growth factor and decreased brain-derived neurotrophic factor in non-Alzheimer's disease tauopathies. Neurobiol Aging. 2014;35:926–33. 24112788 10.1016/j.neurobiolaging.2013.08.029

[50] Harrison JR, Owen MJ. Alzheimer’s disease: the amyloid hypothesis on trial. Br J Psychiatry 2016;208:1–3. 26729836 10.1192/bjp.bp.115.167569

[51] Bretteville A, Planel E. Tau aggregates: toxic, inert, or protective species? J Alzheimers Dis. 2008;14:431–6. 18688094 10.3233/jad-2008-14411

[52] Nakamura M, Shiozawa S, Tsuboi D, Amano M, Watanabe H, Maeda S, et al. Pathological progression induced by the frontotemporal dementia-associated R406W tau mutation in patient-derived iPSCs. Stem Cell Reports. 2019;13:684–99. 31543469 10.1016/j.stemcr.2019.08.011 PMC6829766

[53] Passarella D, Goedert M. Beta-sheet assembly of tau and neurodegeneration in *Drosophila melanogaster*. Neurobiol Aging. 2018;72:98–105. 30240946 10.1016/j.neurobiolaging.2018.07.022 PMC6327151

[54] Funderburk SF, Marcellino BK, Yue Z. Cell “‘self-eating’ (autophagy) mechanism in Alzheimer's disease”. Mt Sinai J Med. 2010;77:59–68. 20101724 10.1002/msj.20161 PMC2835623

[55] Lee JA, Gao FB. Regulation of Abeta pathology by beclin 1: a protective role for autophagy? J Clin Invest. 2008;118:2015–8. 18497881 10.1172/JCI35662 PMC2391068

[56] Ihara Y, Morishima-Kawashima M, Nixon R. The ubiquitin–proteasome system and the autophagic–lysosomal system in Alzheimer disease. Cold Spring Harb Perspect Med. 2012;2:a006361. 22908190 10.1101/cshperspect.a006361 PMC3405832

[57] Rajaguru P, Vaiphei K, Saikia B, Kochhar R. Increased accumulation of dendritic cells in celiac disease associates with increased expression of autophagy protein LC3. Indian J Pathol Microbiol. 2013;56:342. 24441219 10.4103/0377-4929.125282

[58] Vidoni C, Follo C, Savino M, Melone MAB, Isidoro C. The role of cathepsin D in the pathogenesis of human neurodegenerative disorders. Med Res Rev. 2016;36:845–70. 27114232 10.1002/med.21394

[59] Urbanelli L, Emiliani C, Massini C, Persichetti E, Orlacchio A, Pelicci G, et al Cathepsin D expression is decreased in Alzheimer's disease fibroblasts. Neurobiol Aging. 2008;29:12–22. 17049675 10.1016/j.neurobiolaging.2006.09.005

[60] Cataldo AM, Barnett JL, Berman SA, Li J, Quarless S, Bursztajn S, et al Gene expression and cellular content of cathepsin D in Alzheimer's disease brain: evidence for early up-regulation of the endosomal-lysosomal system. Neuron 1995;14:671–80. 7695914 10.1016/0896-6273(95)90324-0

[61] McGrath LT, McGleenon BM, Brennan S, McColl D, McIlroy S, Passmore AP. Increased oxidative stress in Alzheimer’s disease as assessed with 4‐hydroxynonenal but not malondialdehyde. QJM. 2001;94:485–90. 11528012 10.1093/qjmed/94.9.485

[62] Christen Y. Oxidative stress and Alzheimer disease. Am J Clin Nutr. 2000;71:621S–9S. 10681270 10.1093/ajcn/71.2.621s

