# Supplementary material for: Distinctive alteration in the expression of autophagy genes in Drosophila models of amyloidopathy and tauopathy
Source: Ups J Med Sci. 2020 Jul 11;125(4):265–73. doi: 10.1080/03009734.2020.1785063 (PMC7594860; doi:10.1080/03009734.2020.1785063)
Supplement: Supplemental Material [file IUPS_A_1785063_SM4246.docx]

Supplementary data:

**Table S1.** Sequence of the primers

| Gene name | Forward primers | Reverse primers | Basic Tm^1^  (°C) | Ta^2^  (°C) | Ta  (°C) | cDNA (ng) |
| --- | --- | --- | --- | --- | --- | --- |
| RPL32 | TGCTAAGCTGTCGCACAAATGGC | TGTTCGATCCGTAACCGATGTTGGG | F:57.1  R:59.3 | F: 58.79  R:59.45 | 57 | 50 |
| Hook | CGCATTCTTTCCCTGTCGCAT | GGAGTGTCTTTTGTTCATTTAGC | F:54.4  R:51.7 | F: 54.13  R:53.32 | 59 | 75 |
| Atg6 | GGAGTTATCTTTGCCCATC | TAGAGTCCGTAAGCCTGT | F:48.9  R:48.0 | F: 56.4  R:56.13 | 61 | 50 |
| Atg8 | GGTGATTTGGACAAGAAGAAGTA | TCCTCGTGATGTTCCTGGTACGA | F:51.7  R:57.1 | F: 58.92  R:60.54 | 61 | 50 |
| Cathepsin D | ATCATCGGTGGTCAGTATGT | AGAATATAGTCCTTGCCCTGC | F:49.7  R:52.4 | F: 55.17  R:56.05 | 61 | 50 |

- 45 cycles were applied for all primers

1. Tm= 64.9 +41*(yG+zC-16.4)/(wA+xT+yG+zC)
2. Ta of each primer:  Ta = 0.3 x Tm(primer) + 0.7 Tm (product) – 14.9

**Table S2.**  Bayesian hierarchical linear model estimations table for climibing assay based on the median of posterior sampling for each model parameters.

| Model Parameters | n_eff |  | Rhat | mean | sd | HPD 2.5% | HPD  97.5% |
| --- | --- | --- | --- | --- | --- | --- | --- |
| (Intercept) | 4401 |  | 1 | 80.4 | 0.8 | 78.7 | 82 |
| groupT406 | 5261 |  | 1 | 0.8 | 1.1 | -1.5 | 3.1 |
| tm5 Days | 4848 |  | 1 | 13.6 | 1.2 | 11.2 | 15.8 |
| group Aβ42: subgroupCross | 5092 |  | 1 | -34.8 | 1.2 | -37.1 | -32.4 |
| group Tau R406W: subgroupCross | 6215 |  | 1 | -42.5 | 1.1 | -44.7 | -40.2 |
| group Tau R406W:tm5 Days | 5550 |  | 1 | 1.2 | 1.6 | -2.1 | 4.5 |
| group Aβ42: subgroupCross:tm5 Days | 5860 |  | 1 | 4.9 | 1.6 | 1.6 | 8.1 |
| group Tau R406W: subgroupCross:tm5 Days | 7556 |  | 1 | 3 | 1.6 | -0.3 | 6.1 |
| sigma | 2882 |  | 1 | 1.4 | 0.3 | 0.9 | 2.1 |

- * Highest posterior density (HPD) interval a Bayesian Credible Intervals that is estimated based on the quantile of Posterior distribution of each parameter.

**Table S3.** Independed-sample t-test out put for climbing assay

| Flies types | Mean difference (control-cross) | P value | Standard Errors difference (control-cross) | Fold  change |
| --- | --- | --- | --- | --- |
| Aβ42 5days | 29.67 | ≥0.001 | 0.53 | 0.32 |
| Tau R406W 5 days | 39.59 | ≥0.001 | 1.23 | 0.42 |
| Aβ42 25 days | 34.9 | ≥0.001 | 1.14 | 0.44 |
| Tau R406W 25 days | 42.6 | ≥0.001 | 0.59 | 0.53 |

*p value less than 0.05, ** p value less than 0.01, *** p value less than 0.001


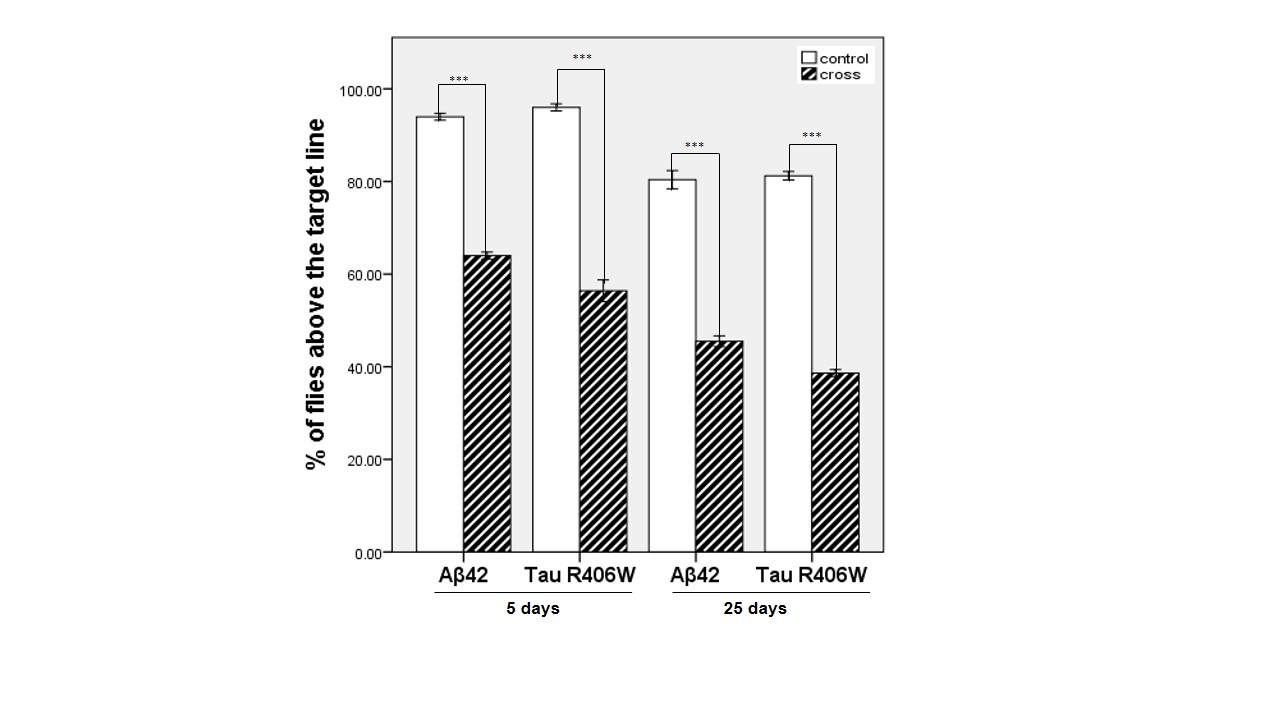


Figure S1: Significant decrease in the climbing ability was observed in 5 and 25-day old flies expressing Aβ42 or Tau R406W. *p value less than 0.05, ** p value less than 0.01, *** p value less than 0.001

**Table S4.**  Bayesian hierarchical linear model estimations table for genes expression based on the median of posterior sampling for each model parameters.

| Model Parameters | n_eff | Rhat | mean | sd | 2.5% | 97.5% |
| --- | --- | --- | --- | --- | --- | --- |
| (Intercept) | 2215 | 1 | 1 | 0 | 0.9 | 1 |
| group Tau R406W | 2111 | 1 | 0 | 0.1 | -0.1 | 0.2 |
| tm5 Days | 2070 | 1 | 0 | 0.1 | -0.1 | 0.2 |
| gene*Atg8* | 2388 | 1 | 0.1 | 0.1 | 0 | 0.2 |
| gene*Atg6* | 2570 | 1 | 0 | 0.1 | -0.1 | 0.1 |
| geneHook | 2408 | 1 | 0 | 0.1 | -0.1 | 0.2 |
| group Aβ42: subgroupCross | 2337 | 1 | -0.7 | 0.1 | -0.8 | -0.6 |
| group Tau R406W: subgroupCross | 2863 | 1 | -0.9 | 0.1 | -1 | -0.7 |
| group Tau R406W: tm5 Days | 2001 | 1 | 0 | 0.1 | -0.2 | 0.1 |
| group Tau R406W: geneAtg*8* | 2431 | 1 | -0.1 | 0.1 | -0.2 | 0.1 |
| group Tau R406W: geneAtg*6* | 2464 | 1 | 0 | 0.1 | -0.2 | 0.1 |
| group Tau R406W: geneHook | 2337 | 1 | 0 | 0.1 | -0.2 | 0.1 |
| tm5 Days: geneAtg*8* | 2365 | 1 | -0.1 | 0.1 | -0.2 | 0.1 |
| tm5 Days: geneAtg*6* | 2526 | 1 | 0 | 0.1 | -0.2 | 0.1 |
| tm5 Days:gene*Hook* | 2408 | 1 | -0.1 | 0.1 | -0.2 | 0.1 |
| group Aβ42: subgroupCross:tm5 Days | 2109 | 1 | 0.6 | 0.1 | 0.5 | 0.8 |
| group Tau R406W: subgroupCross:tm5 Days | 2800 | 1 | 2.1 | 0.1 | 1.9 | 2.2 |
| group Aβ42: subgroupCross: geneAtg*8* | 2624 | 1 | 0.2 | 0.1 | 0.1 | 0.4 |
| group Tau R406W: subgroupCross: geneAtg*8* | 3375 | 1 | 0.2 | 0.1 | 0 | 0.3 |
| group Aβ42: subgroupCross: geneAtg*6* | 2539 | 1 | 0.1 | 0.1 | 0 | 0.3 |
| group Tau R406W: subgroupCross: geneAtg*6* | 3493 | 1 | 0 | 0.1 | -0.1 | 0.2 |
| group Aβ42: subgroupCross:gene*Hook* | 2631 | 1 | 0 | 0.1 | -0.1 | 0.2 |
| group Tau R406W: subgroupCross:gene*Hook* | 3325 | 1 | 2.2 | 0.1 | 2.1 | 2.4 |
| group Tau R406W: tm5 Days: geneAtg*8* | 2446 | 1 | 0.1 | 0.1 | -0.2 | 0.3 |
| group Tau R406W: tm5 Days: geneAtg*6* | 2485 | 1 | 0 | 0.1 | -0.2 | 0.3 |
| group Tau R406W: tm5 Days:gene*Hook* | 2418 | 1 | 0 | 0.1 | -0.2 | 0.2 |
| group Aβ42: subgroupCross:tm5 Days: geneAtg*8* | 2403 | 1 | 2 | 0.1 | 1.8 | 2.2 |
| group Tau R406W: subgroupCross:tm5 Days: geneAtg*8* | 3325 | 1 | 3.9 | 0.1 | 3.6 | 4.1 |
| group Aβ42: subgroupCross:tm5 Days: geneAtg6 | 2431 | 1 | 3.4 | 0.1 | 3.1 | 3.6 |
| group Tau R406W: subgroupCross:tm5 Days: geneAtg*6* | 3393 | 1 | 4.1 | 0.1 | 3.9 | 4.3 |
| group Aβ42: subgroupCross:tm5 Days:gene*Hook* | 2601 | 1 | -0.5 | 0.1 | -0.7 | -0.3 |
| group Tau R406W: subgroupCross:tm5 Days:gene*Hook* | 3224 | 1 | -3.4 | 0.1 | -3.6 | -3.2 |
| sigma | 2528 | 1 | 0.1 | 0 | 0.1 | 0.1 |
| mean_PPD | 10201 | 1 | 1.4 | 0 | 1.4 | 1.4 |
| log-posterior | 1360 | 1 | 50.6 | 8.4 | 33 | 65.7 |

**Table S5.** Independed-sample t-test out put for the levels of *Hook* gene expression

| Flies types | Mean difference (control-cross) | P value | Standard Errors difference (control-cross) | Fold  change |
| --- | --- | --- | --- | --- |
| Aβ42 5days | 0.483 | 0.001 | 0.05 | 0.5 |
| Tau R406W 5 days | -0.066 | 0.485 | 0.080 | 1.06 |
| Aβ42 25 days | 0.583 | 0.002 | 0.078 | 0.59 |
| Tau R406W 25 days | -1.24 | 0.018 | 0.174 | 2.26 |

**Table S6.** Independed-sample t-test out put for the levels of *Atg6* gene expression

| Flies types | Mean difference (control-cross) | P value | Standard Errors difference (control-cross) | Fold  change |
| --- | --- | --- | --- | --- |
| Aβ42 5days | -3.41 | ≥0.001 | 0.090 | 4.45 |
| Tau R406W 5 days | -5.35 | ≥0.001 | 0.082 | 6.31 |
| Aβ42 25 days | 0.57 | ≥0.001 | 0.012 | 0.58 |
| Tau R406W 25 days | 0.83 | ≥0.001 | 0.016 | 0.84 |

**Table S7.** Independed-sample t-test out put for the levels of *Atg8* gene expression

| Flies types | Mean difference (control-cross) | P value | Standard Errors difference (control-cross) | Fold  change |
| --- | --- | --- | --- | --- |
| Aβ42 5days | -2.16 | ≥0.001 | 0.035 | 3.15 |
| Tau R406W 5 days | -5.23 | ≥0.001 | 0.12 | 6.28 |
| Aβ42 25 days | 0.45 | ≥0.001 | 0. 032 | 0.45 |
| Tau R406W 25 days | 0.68 | ≥0.001 | 0.023 | 0.70 |

**Table S8.** Independed-sample t-test out put for the levels of *Cathepsin D* gene expression

| Flies types | Mean difference (control-cross) | P value | Standard Errors difference (control-cross) | Fold  change |
| --- | --- | --- | --- | --- |
| Aβ42 5days | 0.013 | 0.89 | 0.097 | 0.03 |
| Tau R406W 5 days | -1.26 | 0.007 | 0.13 | 2.24 |
| Aβ42 25 days | 0.68 | ≥0.001 | 0.026 | 0.74 |
| Tau R406W 25 days | 0.84 | ≥0.001 | 0.03 | 0.85 |


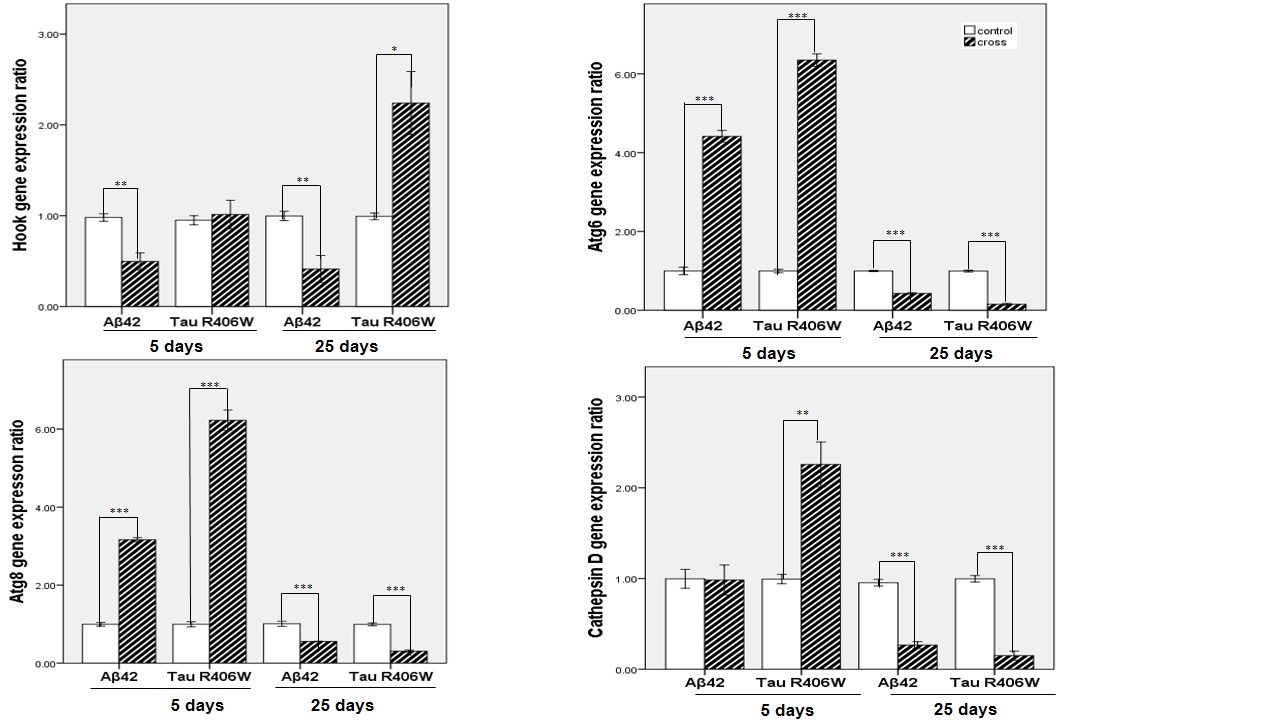


Figure S2: Fold change analysis for autophagy gene expressions in Aβ42 Tau R406W expressing flies. *p value less than 0.05, ** p value less than 0.01, *** p value less than 0.001
